# Regulation of the promoter for capsular polysaccharide synthesis in *Neisseria meningitidis* serogroup B by HTH_XRE family transcription factor

**DOI:** 10.1128/spectrum.03301-24

**Published:** 2025-04-24

**Authors:** Iyinoluwa Sofowora, Pumtiwitt McCarthy, James Wachira

**Affiliations:** 1 Department of Biology, Morgan State University, Baltimore, Maryland, USA; 2 Department of Chemistry, Morgan State University, Baltimore, Maryland, USA; Griffith University - Gold Coast Campus, Gold Coast, Queensland, Australia

**Keywords:** HTH_XRE transcription factor, *cis*-regulatory elements, capsular polysaccharides synthesis, two-component regulatory systems, *Neisseria meningitidis*, *misR/misS*, NusG, CrgA, transcription factors, RpoD

## Abstract

The capsular polysaccharide synthesis (*cps*) locus of *Neisseria meningitidis* is implicated in invasive meningococcal disease. The synthesis (*synABCD*) and transport (*ctrABCD*) operons are transcribed in opposite directions from a common intergenic region, and the expression is negatively regulated by the bacterial two-component system (TCS) *misR/misS* and thermosensitive RNA folding. However, these mechanisms do not fully explain the stationary phase responses, and the *cis*-acting elements remain to be fully characterized. Using the GFP reporter gene and site-directed mutagenesis, *cis*-regulatory elements in the 134 bp intergenic region, NmIR, were investigated. While confirming a known RpoD promoter, an additional potential promoter element and putative binding sites for the transcription factors *fis and lexA* were identified through sequence analysis. Deletion of the putative *lexA* binding site led to an increase in GFP fluorescence. The *N. meningitidis* genome carries only one *lexA* homolog, the helix-turn-helix regulator XRE family member (GenBank-NMB0910, HTH_XRE). Trans-complementation of the NmIR-GFP reporter with the *N. meningitidis* HTH_XRE expression plasmid led to increased fluorescence. Trans-complementation with either *misR/misS* or *nusG* decreased reporter gene expression. Consistent with previous reports, deletion of the RpoD promoter reduced expression by 50%, suggesting the redundancy of promoter elements in the intergenic region. Thus, the results confirm the functioning of an exogenous *N. meningitidis* capsule synthesis promoter in *Escherichia coli* and demonstrate its regulation through trans-complementation by *misR/misS*, HTH_XRE, and *nusG*.

**IMPORTANCE:**

Pathogenic *Neisseria meningitidis*, a causal agent of bacterial meningitis, secretes capsular polysaccharides of different compositions that differentiate individual serogroups. Since the capsule is an important virulence factor that determines adhesion to epithelia and ability to invade tissues, there is a need to understand the underlying mechanisms for capsule expression. Furthermore, bacterial polysaccharides are potential sources of novel biomaterials. The expression of the capsule production genes is regulated, and this study reveals a mechanism involving a transcription factor, HTH_XRE, whose function in *N. meningitidis* is not known. It extends the understanding of capsular expression regulation by identifying other control elements in the intergenic region. The results will have applications in optimizing bacterial biomaterials production or in developing therapeutic interventions.

## INTRODUCTION

The *Neisseria meningitidis* capsule is implicated in virulence, and it is a target for meningococcal vaccines (1, 2). Among the identified 12 serogroups that are distinguished by the structure and composition of capsular polysaccharides, six (A, B, C, W, X, and Y) are known to cause invasive disease (2, 3). Serogroups B, C, W, and Y produce sialic acid-containing capsules, while serogroups A and X produce *N*-acetyl-d-mannosamine-1-phosphate and *N*-acetyl-d-glucosamine-1-phosphate containing capsules, respectively (4). Still, the organization of the capsule synthesis (*cps*) loci in these and many other serogroups is highly conserved (4). In the sialic acid-producing serogroups, it includes an operon that codes for sialic acid synthesis enzymes (*synABC/cssA-C*) and polysialyltransferase (*synD*/*cssD*) and an oppositely oriented capsule transport operon that consists of four genes *ctrA-D* (5). The promoters for both the synthesis and transport operons are contained in a 134 bp intergenic region (NmIR) (6). Capsular expression is regulated by several mechanisms, including transcriptional and translational mechanisms (6).

The regulatory elements for the synthesis operon were initially identified as an UP-like element located next to the −35-promoter region and a yet unidentified negative element contained in the +13 to +103 region (7). Deletions encompassing the UP-like element and the −35 box reduced reporter gene activity by about 50% (7), suggesting the presence of other elements with promoter activity. Further, environmental conditions such as temperature, glucose concentration, and iron concentration did not affect capsule production (7). However, the 5′-UTR region that includes the direct repeat in the NmIR and a segment that includes the ribosome binding site (RBS) has the potential to form a stable hairpin that is proposed to regulate translation in some strains in a temperature-sensitive manner (6). Given the discrepancies in literature reports, the role of temperature in regulating capsule expression in *N. meningitidis* warrants further study.

The UP-like element is juxtaposed to the −35-promoter box on the 5′ end, and another study identified a contact-regulated gene A (*crgA*)/*lysR* transcription factor-binding site on the 3′ side that overlaps it (8). The CrgA binding site is a negative regulatory element that functions to downregulate *syn* genes during adhesion to epithelial cells, but not during growth in suspension (8). The *crgA* gene is upregulated during coculture with epithelial cells together with the regulatory protein *misR*/*phoP*, a two-component global regulatory system protein that is also implicated in the regulation of capsule expression (9). Mutation of the *misR/misS* is associated with increased capsule expression, and MisR binds to DNA encompassing NmIR and the 5′-coding region of *cssA*, the first gene in the capsule synthesis operon (6, 10). MisR was demonstrated to bind to 14 promoters within the genome, including the promoters for three transcription factors that carry the helix-turn-helix (HTH) DNA binding domain (6, 11, 12).

In this study, a negative regulatory element with sequence homology to LexA binding sites was identified in the direct repeat sequence. HTH_XRE, an *N. meningitidis* LexA homolog, upregulated NmIR-dependent transcription.

## RESULTS

### Identification of putative binding sites and promoters

**Fig 1:**
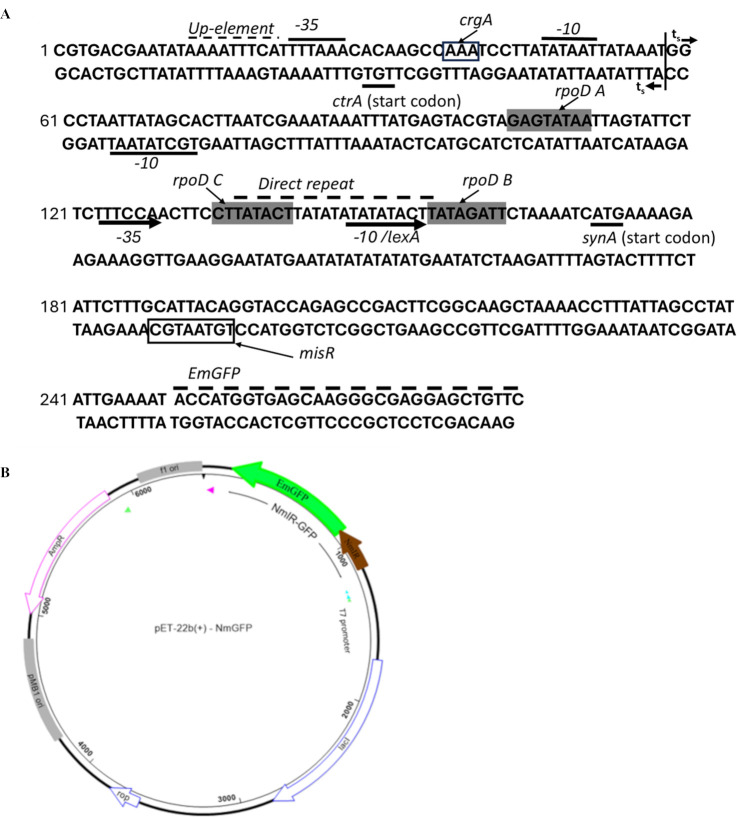
Regulatory elements in NmIR. (**A**) Experimentally determined and predicted elements in the intergenic and flanking sequences are boxed. The known promoter elements, transcription start sites (ts), and coding regions are as reported in the literature (see text for details). The CrgA binding site is also as reported in the literature. (**B**) Map of the promoter construct used in this study. The NmIR sequence (highlighted in brown) includes the full promoter region and the first 26 amino acids of the *synA* open reading frame fused to the GFP coding sequence. In DH5alpha, the GFP is therefore expressed from the syn promoter and translational signal.

Softberry BPROM software (13) analysis of NmIR identified putative binding sites for the TFs *fis and lexA* and three *rpoD* sites (*rpoD A*, *rpoD B*, *and rpoD C*) that are calculated to be significantly homologous to the −10-promoter box (Table 1; Fig. 1). Two promoters were identified: one adjacent to the UP-like element, and that is already experimentally determined based on the literature, and one next to the direct repeat region. These predicted promoters with consensus −10 box (TTATATACT) and a −35 box (TTTCCA) were identified upstream of the *synA* gene (GenBank accession number: X78068.1). *misR/misS* has been reported to be a negative regulator of capsule expression in *N. meningitidis* (10, 11). Sequence alignments of reported MisR consensus sequences were used as input for FIMO software leading to the prediction of a putative binding site for MisR within the *synA* gene (Fig. 1). Previous reports have demonstrated the binding of MisR to a 600 bp DNA fragment encompassing the intergenic region and including the flanking coding regions of *synA* (10).

**TABLE 1:** BPROM promoter prediction^*a*^

TF	Score	Sequence	Location relative to the initiation codon
*rpoD* (−10)	9	GAGTATAA	−59
*rpoD* (−10)	10	CTTATACT	−28
*fis*	18	TATACTTA	−26
*lexA*	14	TATATATA	−20
*lexA*	14	TATATATA	−18
*lexA*	14	TATATATA	−16
*lexA*	12	ATATATAC	−15
*lexA*	18	TATATACT	−14
*fis*	18	TATACTTA	12
*rpoD* (−10)	11	TATAGATT	−6

^*a*^ A 249-sequence encompassing the initial coding regions of *ctrA* and *synA* was used to detect promoter elements.

### Functional analysis of NmIR in *E. coli*

**Fig 2:**
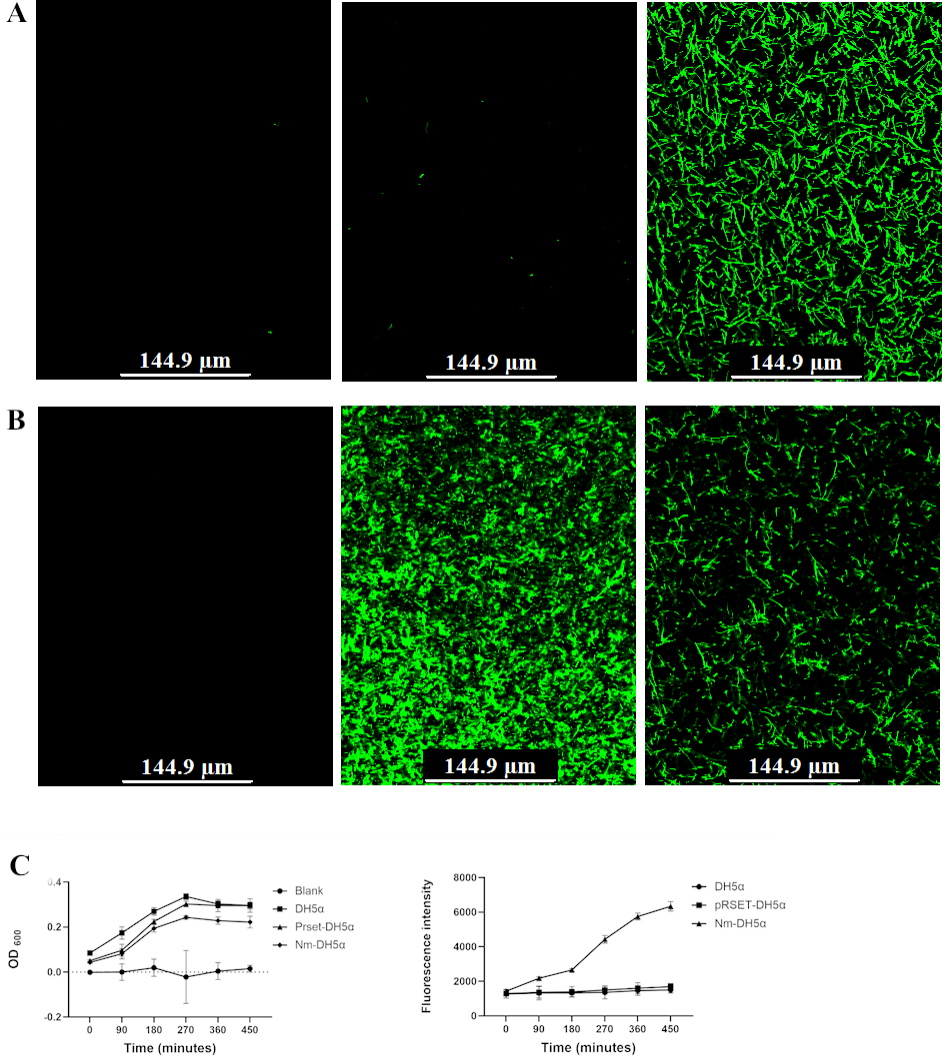
Functional analysis of GFP expression from NmIR. (**A**) Comparison of GFP fluorescence in *E.coli* DH5α. Untransformed DH5α background control (left panel); pRSET-EmGFP transformed DH5α (middle panel); and NmIR-GFP-pRSET transformed DH5α (right panel) constructs expressed in DH5α. (**B**) Comparison of GFP fluorescence in *E.coli* BL21(DE3). Untransformed BL21(DE3) (left panel); pRSET-EmGFP transformed BL21(DE3) (middle panel); and NmIR-GFP-pRSET transformed BL21(DE3) (right panel). No fluorescence was detected in the pRSET-EmGFP transformed DH5α, while fluorescence was detected in the BL21(DE3) cells. In contrast, the NmIR-GFP-pRSET construct is fluorescent when expressed in *E. coli* DH5α and BL21(DE3) strains. (**C**) Expression curve of Nm as a function of the growth stage. Optical density (OD) and GFP fluorescence were quantified using the BioTek microplate reader.

The studies were conducted in *Escherichia coli. N. meningitidis* capsular genes can be exogenously expressed in *E. coli* from their natural promoter, with greater expression being seen at the stationary phase (14). This was recapitulated by the NmIR-GFP-pRSET construct (Fig. 2). NmIR-GFP-pRSET is fluorescent in *E. coli* DH5α and BL21(DE3) strains, whereas the pRSET vector alone without NmIR is only fluorescent in BL21(DE3) strains, whereby it is expressed from the T7 promoter. Elevated expression is observed as cultures transition to the stationary phase (Fig. 2C).

### Role of predicted control elements in capsular expression

**Fig 3:**
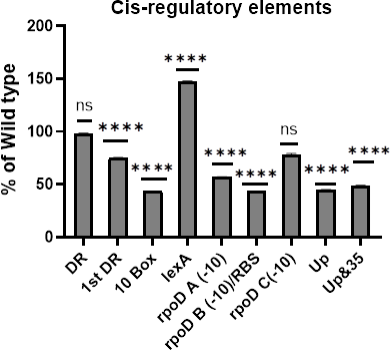
*Cis*-regulatory elements in the NmIR. Elements identified through sequence analysis were deleted, and the effect on promoter activity as measured through GFP fluorescence was determined. Deletion of the −10-promoter region, *rpoD* binding sites A and B, and UP-like element led to a 50% reduction in capsular expression, whereas deletion of the *lexA* (the predicted −10 promoter) site led to a 47% increase in capsular expression. Statistical significance relative to the wild type was analyzed by one-way ANOVA followed by Dunnett's multiple comparisons test (n = 5). ****, *P* < 0.0001 and ns, *P* > 0.05 .

Deletions of the −10-promoter box, UP-like element, UP-like element, and −35 box, *rpoD* A, and *rpoD* B regions of NmIR individually resulted in an approximately 50% reduction in GFP expression. Deletion of the *lexA* region that is also predicted to encode a −10-box led to a 47% increase in GFP expression. Deletion of the first repeat sequence led to a 15% decrease in GFP expression. Deletion of the *rpoD* C element and the direct repeat had no significant effect on promoter activity, as shown in Table 2 and Fig. 3.

**TABLE 2:** *Cis*-acting elements regulating the *synABCD* operon^*a*^

NmIR mutation	Mean fluorescence intensity (arbitrary units)	% WT	Standard deviation	*P* value
WT	2.61	100	0.43	control
DR	2.61	100.02	0.36	>0.9999
1st DR	1.96	75.122	0.25	<0.0001
10 box	1.13	43.54	0.18	<0.0001
*lexA*	3.84	147.32	0.61	<0.0001
*rpoD A* (−10)	1.48	56.84	0.30	<0.0001
*rpoD B* (−10)/RBS	1.13	43.53	0.11	<0.0001
*rpoD C* (−10)	2.52	96.53	0.24	0.9785
Up	1.17	44.90	0.13	<0.0001
Up and −35	1.27	48.90	0.27	<0.0001

^*a*^ The predicted elements were deleted though site-directed mutagenesis and GFP fluorescence intensity determined. Image analysis was conducted with ImageJ (15).

### Role of RNA secondary structure

**Fig 4:**
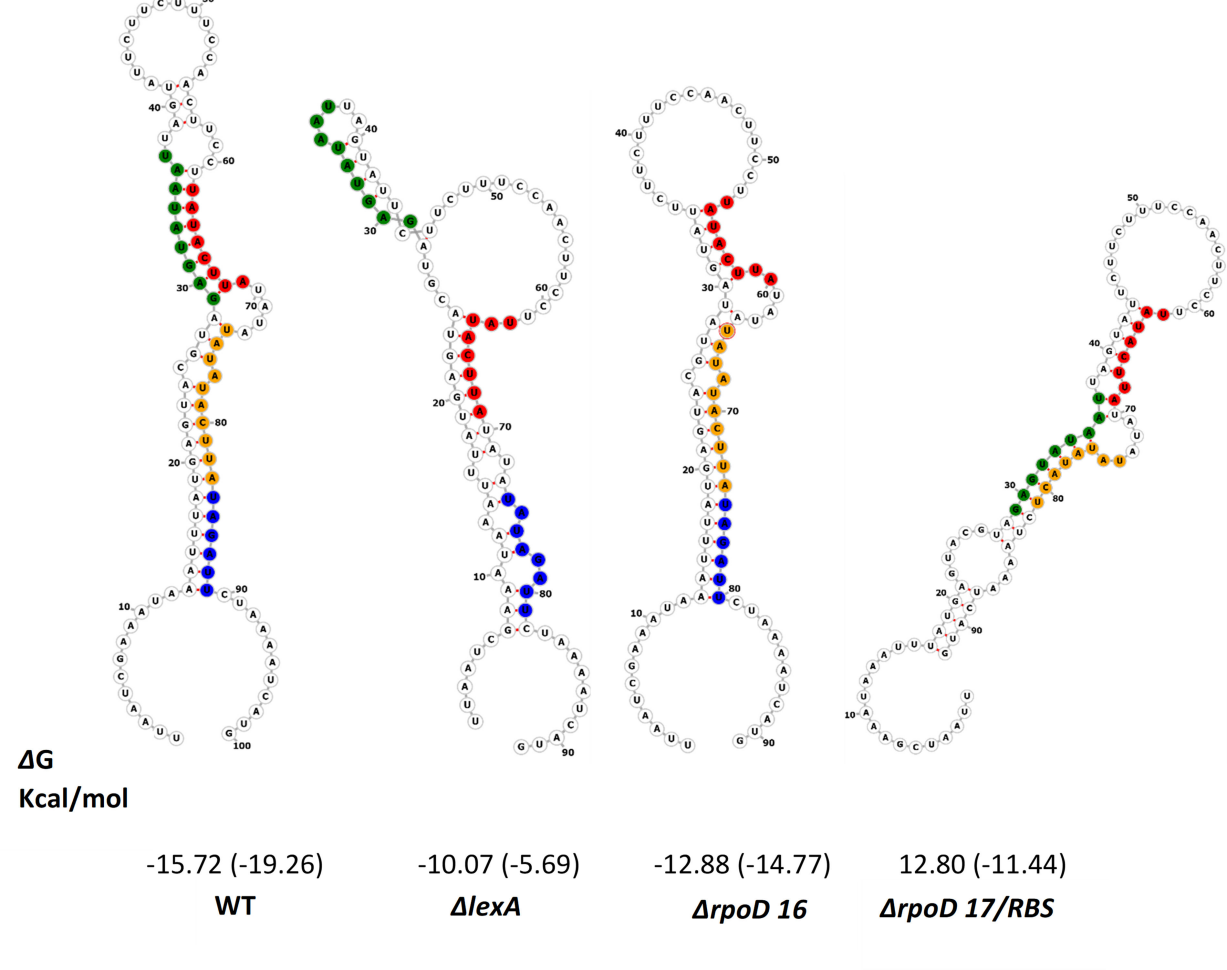
Predicted secondary structures of the 5′-untranslated region of NmIR. The structures of the wild-type (WT) and of the ΔlexA, *ΔrpoD* A, and *ΔrpoD* B/RBS mutants were predicted with RNAfold. The potential regulatory elements are highlighted as follows: The first copy of the direct repeat (red), the second copy of the direct repeat/lexA site (orange), the *rpoD A* site (green), and the ribosomal binding site, which overlaps with the *rpoD B* site (blue). The free energies (ΔG) in kcal/mol are shown below each structure.

Among the three mechanisms of capsule expression that impinge on the NmIR are translational control mediated by secondary structure formation in the 5′-UTR (6). The deletion mutations were analyzed with RNAfold (16). The *lexA* deletion led to apparent destabilization of the secondary structure based on the free energy (−15.72 kcal/mol for the wild type as opposed to −10.07 kcal/mol for the mutant) (Fig. 4).

### Putative *cis*-regulatory elements in the promoter for capsule synthesis operon *in N. meningitidis B*

The intergenic region in sialic acid-producing *N. meningitidis* serogroups (B, C, W, and Y) is conserved, with differences being observed only in the repeat sequence (TATACTATACTTA), which varies between serogroups. Analysis of similar regions in *E. coli* and *Haemophilus influenzae* using sequence alignments revealed the conservation of AATAAAAG and TATATACT. These sequences are putative binding sites for *phoB*, *rpoD*, and *lexA*. This finding is consistent with the data showing the loss of expression of GFP when the *rpoD* site in the region was mutated. Expression of GFP was elevated when the *lexA* site was mutated, suggesting the presence of a negatively acting element.

### Identification of conserved transcription factors involved in capsular synthesis in ABC transporter-dependent microbes

Comparative genomic analysis was conducted to elucidate the evolutionary relationships and potential regulatory mechanisms involved in capsule synthesis. Basic Local Alignment Search Tool (BLAST) analysis revealed that two of the three TFs (*typA* and *nusG*) required for capsular synthesis in *E. coli* are conserved in *N. meningitidis*, whereas the *mprA* homolog was not detected.

**Fig 5:**
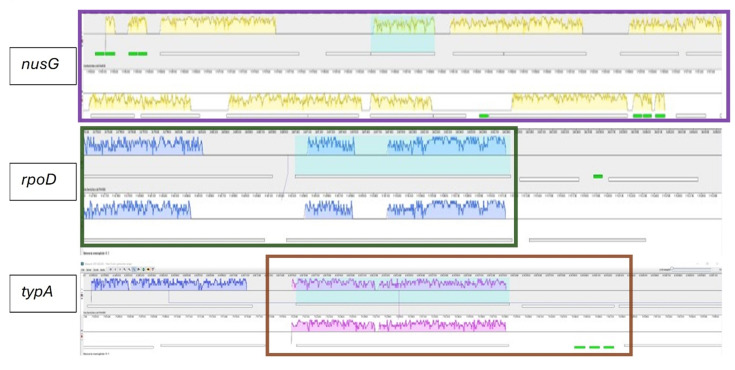
Conservation of genomic regions encoding transcription factors involved in capsule expression in *N. meningitidis B* and *E. coli K1*. MAUVE alignment of *N. meningitidis* MC58 and *E. coli* K1 revealed that nusG (highlighted with a purple box), rpoD (highlighted with a green block), and typA (highlighted with an orange box) are in locally collinear blocks (LCBs) with several translational proteins and some tRNAs in both Nm and *E. coli*, suggesting their functional importance and evolutionary conservation. Blocks of the same color indicate regions of conservation between the two genomes.

The genome alignment of *N. meningitidis MC58* and *E. coli K1* via the Mauve alignment tool revealed that *the nusG*, *rpoD*, and *typA* genes are in locally collinear blocks (LCBs) with several translational proteins and some tRNAs (colored in green) in both organisms (Fig. 5). The LCBs visualized by colored blocks in the alignment highlight areas of high sequence similarity and evolutionary conservation. The co-localization of these TFs within the LCB supports the hypothesis they serve similar functions in both organisms. The association with translational proteins and tRNAs further suggests that the capsular genes are translationally regulated.

### Trans-complementation assays

**Fig 6:**
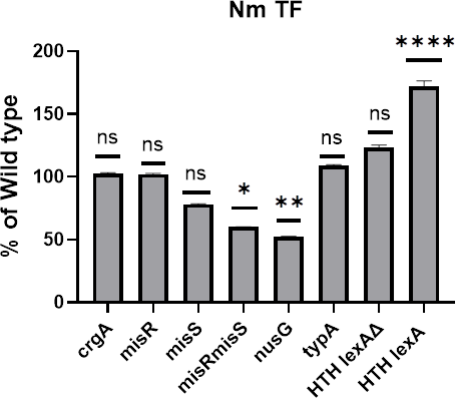
Trans-complementation assays of NmIR with transcription factors involved in capsule regulation. Plasmid constructs of the indicated transcription factors were co-transformed with the NmIR construct into *E. coli* strain DH5α. The bars represent the mean relative fluorescence as a percentage of the NmIR WT alone (% WT). Statistical significance relative to the wild type was analyzed by one-way ANOVA followed by Dunnett's multiple comparisons test (n = 5). ****, *P* < 0.0001; **, *P* < 0.01; *,*P* < 0.05 and ns, *P* > 0.05 .

Co-transformation of NmIR-GFP expression plasmid with HTH regulator expression plasmid led to increased promoter activity, which was not observed when the LexA binding site was deleted (Fig. 6). Conversely, co-transformation with MisR/MisS and NusG expression vectors reduced GFP expression, suggesting roles as repressors. CrgA and TypA expression plasmids had no significant effect on GFP fluorescence (Table 3).

**TABLE 3:** Transcription factors regulating the *synABCD* operon promoter^*a*^

TFs	Mean fluorescence intensity (arbitrary units)	% WT	Standard deviation	*P* value
WT	3.95	100	0.78	control
*crgA*	4.04	102.44	0.75	>0.9999
*misR*	4.06	102.99	0.72	>0.9999
*misS*	3.08	78.14	0.54	0.4838
*misR/misS*	2.40	60.89	0.29	0.0328
*nusG*	2.07	52.33	0.50	0.005
*typA*	4.32	109.35	0.56	0.9859
*HTH_XRE plus ∆lexA*	4.88	123.75	2.25	0.5162
*HTH_XRE*	6.80	172.39	4.36	<0.0001

^*a*^ Transcription factors with the potential to regulate the *synABCD* operon were cloned into a pET22b compatible plasmid, plasmid pstV28, and co-transformed NmIR-GFP-pET22b into *E. coli* DH5α. Fluorescence intensity was measured with confocal microscopy. Image analysis was conducted with ImageJ (15).

## DISCUSSION

*N. meningitidis* serogroups B, C, Y, and W express sialic acid-containing capsules that are synthesized by transport and synthesis operons that are separated by a 134 bp central region that harbors the respective promoters (6). Of note, the operons are transcribed in opposite directions with the transcription start sites being separated by 1 bp, the promoter for the transport operon is devoid of a −35 motif, and a direct repeat sequence in the 5′-UTR of the synthesis operon is predicted to form a stable hairpin that is postulated to regulate translation by sequestering the RBS (6). In addition, the region contains regulatory elements and a MisR binding site, but the precise transcription factor binding site (TFBS) has not been identified (6). This study investigated potential regulatory elements associated with the promoter for the capsule synthesis operon in *N. meningitidis B* MC58 (ATCC 53415D-5).

Multiple potential binding sites for the TFs Fis and LexA and three for sigma^70^ (*rpoD*) were predicted using BPROM software that uses linear discriminant function (13). A promoter associated with these TFBSs was predicted with the TSS coinciding with the “A” of the initiation codon implying a leaderless mRNA. Leaderless mRNAs are present in all domains of life, and in bacteria, they can comprise a significant number of mRNAs, for example, 34% in some *Firmicutes,* 17% in some *Alphaproteobacteria*, and 8% in some *Gammaproteobacteria* (17). Leaderless mRNAs are implicated in adaptation to stress (17). Surprisingly, the known promoter was not detected, although a 249 bp fragment encompassing sections of the coding regions of *ctraA* and *synA* (*synX*) was used in the prediction. However, BPROM has 80% specificity and sensitivity for *E. coli* sigma^70^ promoters (13). The known promoter was mutated in other studies and shown to reduce transcription by twofold (7), suggesting the presence of an additional promoter. Thus, systematic deletion using site-directed mutagenesis was undertaken in this study.

To aid in the study of the activity of the promoter for the capsule synthesis operon under different conditions and growth phases, an NmIR-GFP construct was created via directional cloning. Given the *E. coli* background of the studies, a compatible plasmid was used to co-express the potential transcription factors. NmIR was shown to be active in *E. coli* with increased GFP expression at the stationary phase (Fig. 2), which is consistent with the published literature (7, 14, 18). Indeed, upregulation of capsule expression based on the growth phase is seen in other bacteria (19). The deletion of the identified UP-like element preceding the −35 box resulted in a twofold reduction in capsular expression, and further deletion to include the −35 box did not exacerbate this effect. This finding is consistent with the study of von Loewenich et al. who showed that the deletion of the UP element reduced expression by 50%, but further deletion of the −35 and −10 boxes did not lead to further reduction in reporter-gene expression (7). In contrast, this study revealed that deleting the −10 box alone led to a 57% decrease in expression, highlighting the significant role of the −10 promoter in transcription initiation (Table 1). The NmIR fragment is predicted to contain three *rpoD* sites (Table 1), and the deletion of two of them (*rpoD A and rpoD* B) resulted in more than 50% reduction in promoter activity, suggesting a function in capsule synthesis. The predicted *rpoD B* site coincides with the −10 region of a putative second promoter (Fig. 1). However, this site would produce an mRNA without a 5′-UTR, which requires confirmation through additional experimentation. The genome of MC58 contains three sigma factors (*rpoD*, *rpoH*, and *rpoN*) (20) and only RpoD binding sites are predicted in the *cps* locus intergenic region.

RpoD (sigma^70^) is a conserved sigma factor that controls transcription in bacteria during the exponential growth phase (21). A study by Yin et al. demonstrated that the competition between σ^70^ and other sigma factors for binding sites to RNA polymerase is important for regulating alginate production, with the alternative, stress-related sigma factors leading to enhanced production of this capsular polysaccharide in *Pseudomonas aeruginosa* (22). The use of dual promoters in capsule expression has also been reported in *Staphylococcus aureus,* whereby one dominant promoter under the control of SigB sigma factor is located downstream of a SigA-dependent promoter, and the two promoters are regulated by different mechanisms (19). While parallels cannot be drawn at this time, it is known that *S. aureus* capsule production is subject to regulation by complex interactions between the SaeRS bacteria two-component regulatory system (TCS) and transcription factors that act on competing promoters (23, 24). Similarly, alginate production in *P. aeruginosa* is regulated by the KinB/AlgB TCS with KinB being the sensor kinase and AlgB being the transcription regulator that influences promoter function (25). The TCS is also crucial to capsule expression in *N. meningitidis* capsule expression with MisS functioning as the sensor kinase for the regulator MisR (26). While MisR binds directly to DNA, interaction with specific promoter elements has not been reported. Indeed, sequence analysis identified the potential MisR binding site within the *syn*A coding region (Fig. 1). In these bacteria, the TCS appears to exert negative regulation, which is confirmed in this study for MisS/MisR (Table 3).

Bartley et al. reported the binding of MisR to NmIR and noted the upregulation of both the *ctrA* and *synA* genes in *misR* deletion mutants (10). In another study, the upregulation of capsule expression was reported in *misR*/*misS* mutants that was accompanied by upregulation of *ctrD*, the gene for the capsule export ATP-binding protein (11). The co-transformation of the *misR/misS* genes with NmIR reduced GFP expression, indicating the negative regulatory function of MisR in capsule expression.

The conservation of the *lexA* binding sites in ABC transporter-dependent *cps* loci despite the absence of the *lexA* gene in *N. meningitidis,* as previously reported by Davidsen & Tønjum (27), suggests functional significance (Fig. 5). Deleting this site led to a 47% increase in capsule expression, suggesting a potential regulatory role of a *lexA*-like factor. This is consistent with other studies that showed that the deletion of a 46 bp region encompassing the direct repeats or substitution mutations that disrupted the symmetry increased reporter gene activity (14). However, the sequence used in this study differs from the one reported in the literature (14) in that the direct repeats are interrupted TAT (Fig. 1). Nonetheless, the putative LexA binding site is preserved in both sequences. Thus, a BLAST search of the MC58 genome was conducted with the *E. coli lexA* gene sequence, and HTH_XRE was identified as a homolog. Cloning of HTH_XRE and subsequent trans-complementation with NmIR-GFP led to a 72% increase in GFP expression. In contrast, no change in expression was observed when the *lexA* site was absent, indicating the specificity of this interaction. This result highlights a novel regulatory mechanism. Further studies are needed to characterize the regulatory network involving the HTH_XRE and its potential impact on adhesion or pathogenesis. Given that the assays were conducted in *E. coli*, experiments in *N. meningitidis* will be necessary to confirm the physiological significance of the observation.

Other TFs studied were Fis, because of the identified binding site (Table 1), CrgA, which has been implicated in the downregulation of capsule during adhesion to epithelial cells, and TypA and NusG because of their implication in capsule expression from the analogous *E. coli* operon (8, 28). Fis (a factor for inversion stimulation) is a small basic protein that is important in bacterial nucleoid assembly and recombination processes, and it is also involved in the regulation of many genes, including those involved in two-component systems and lipopolysaccharide biosynthesis (29, 30). The deletion of the repeat region, which spans the *fis* binding site, did not affect capsule expression, as measured by GFP fluorescence. Similarly, *CrgA*, a LysR-type transcriptional regulator, did not influence promoter activity, which is consistent with the findings of von Loewenich et al. (7). This result notwithstanding, a CrgA binding site that overlaps the −35-promoter region has been identified (8). The caveat to these results is that they were conducted in *E. coli*, and the regulation by *crgA* and *misR* appears to be related to *N. meningitidis* adhesion to epithelial cells (8, 9).

In *E. coli*, three TFs (NusG, TypA, and MprA) are important for the transcriptional control of capsule synthesis (28). Comparative analysis revealed that the loci for the *nusG* and *typA* genes, including adjacent sequences, are conserved in *E. coli* and *N. meningitis*. Both *N. meningitidis nusG* and *typA* were cloned and used in trans-complementation studies. NusG is a universally conserved TF that binds to RNA polymerase to drive RNA synthesis and influence Rho-dependent termination (31). It is known to function as both a terminator and an anti-terminator (31, 32). The observed reduction in GFP expression suggests that in the *N. meningitidis* capsule synthesis operon, NusG primarily acts as a transcription terminator. There was no significant effect of *typA* on GFP expression upon trans-complementation with NmIR.

### Conclusion

A GFP fluorescence reporter plasmid was used to further delineate the mechanisms regulating *N. meningitidis* capsule expression promoter. Novel TFBSs were identified, thereby extending the known mechanisms that involve MisR/MisS and CrgA. HTH_XRE, a *N. meningitidis* gene with unknown function was identified as a potential factor regulating capsule production. This factor potentially regulates the downstream promoter for leaderless mRNA. Leaderless mRNAs correlate with stress, and results presented in this study indicate the stimulation of GFP expression during stationary phase. Therefore, similar to other bacterial pathogens, the regulation of capsule expression appears to involve multiple mechanisms and TFs. These studies will have the utility of enhancing the production of capsular polysaccharides-based biomaterials.

## MATERIALS AND METHODS

### Materials

*N. meningitidis* serogroup B strain genomic DNA (53415D-5) was obtained from ATCC (Manassas, VA). Plasmid pET-22b(+) was purchased from EMD Millipore. Plasmid pSTV 28 DNA was purchased from Takara Bio USA, Inc., and it was used as a vector for transcription factors. Plasmid pSTV 28 carries a pACYC184 origin and the chloramphenicol resistance gene for Tn9. It is compatible with the pET-22b(+) vector allowing for trans-complementation studies. Bacteria culture medium (terrific broth) was purchased from Midwest Scientific, Fenton, MO. The following were undertaken with kits and supplies from New England Biolabs, Ipswich, MA: site-directed mutagenesis was carried out with Q5 Site-Directed Mutagenesis Kit; clean-ups of PCR and restriction enzyme digests were undertaken with Monarch PCR & DNA Cleanup Kits; ligation was undertaken with Quick Ligation Kit; restriction enzyme digests followed NEB’s recommended protocols; and BL21(DE3) and DH5α (NEB 5-alpha) competent *E. coli* cells were used for transformations. In some cases, cloning was accomplished with In-Fusion Snap Assembly Master Mix, Takara Bio USA, Inc. Plasmid purifications were undertaken with QIAprep Spin Miniprep Kit (Qiagen LLC, Germantown, MD) or Zyppy Plasmid Miniprep Kit, Zymo Research Corporation. Custom PCR primers were obtained from Integrated DNA Technologies, CORALVILLE, IA. Confocal Microscopy was conducted with a Leica STELLARIS 5 Confocal Microscope (Leica Microsystems Inc., Deerfield, IL). Imaging was conducted in µ-Slide 8-Well glass bottom chambers with #1.5H glass bottom coverslip (Ibidi, 80827).

### Bioinformatic analysis

#### Regulatory elements prediction

The relevant *N. meningitidis* serogroup B genomic sequence was retrieved from the National Center for Biotechnology InformationGenBank (X78068.1). The sequence encompassing the NmIR and the region encoding the first 26 amino acids of SynA was analyzed for the presence of putative TFBSs with BPROM from Softberry (13).

#### Sequence alignments

To identify conserved sequences, the corresponding NmIR regions from other *N. meningitidis* serogroups were also retrieved from GenBank and aligned with Clustal Omega through the EMBL-EBI Job Dispatcher (33)

#### Identification of transcription factor sequences

Transcription factors that are known to regulate capsule expression in *E. coli* K12 were used to identify *N. meningitidis* homologs. The BLAST, tblastn, was used to identify homologous genes in the genome *of N. meningitidis* strain MC58 (serogroup B).

#### Visualization of alignments

The alignments were visualized with JalView (34). WebLogo was used to generate a visual of conserved residues and their positions based on the alignments (35).

#### Identification of syntenic genome regions

Mauve software, a multiple genome alignment tool, was used to determine the LCBs in the respective chromosomal regions (36). The binding sites of the TFs in the intergenic region were mapped via FIMO software (37).

### Construction of reporter vectors

#### Construction of a reporter strain with *Nm* intergenic region (*NmIR*) promoter activity

The NmIR region, including the first 78 nucleotides of the *synA* reading frame, was cloned upstream of the GFP reading frame to generate a fusion protein that includes the first 26 amino acid residues of *synA*. The fragment also includes the first 30 nucleotides of the ctrA reading frame. The NmIR was PCR amplified by designing primers that incorporated restriction enzyme sites *Bam*H1 (5′-TTT GGA TCC GTG ACG AAT ATA AAA TTT CAC TTT-3′ and *NCO*I (5′-A CAC CAT GGT ATT TTC AAT ATA GGC TAA TAA AGG TTT TAG CTT-3′) at the ends, allowing the directional cloning of the PCR product into the plasmid expression vector pRSET-EmGFP (Thermo Fisher Scientific, Waltham, MA). The cassette containing NmIR fused to EmGFP was then transferred to the *Bam*H1/*Hin*dIII site of pET-22b(+), which had been double digested with *Bam*H1 and *Hin*dIII restriction enzymes (Invitrogen). The digested vector and PCR product were ligated and transformed into competent *E. coli* DH5α cells, yielding NmIR-GFP. The GFP coding region was similarly transferred to the pET-22b(+) to serve as a control. The desired plasmid constructs were identified via a panel of colony PCRs and confirmed through Sanger sequencing through the Genetic Resources Core Facility at Johns Hopkins University School of Medicine.

#### Construction of NmIR mutants and GFP fluorescence measurement

Q5 Site-Directed Mutagenesis Kit used to create deletions of the identified binding sites and the TFs in the NmIR. NEB base changer v1 primer design software was used to design primer pairs for the respective TFBSs. The mutations and primers are listed in Table S1, with the deleted sequence in bold. The different regions were amplified, yielding a linear product missing the region deleted. The enzyme *DpnI* (KLD) was used to digest the template, and the linear product was ligated to a circular version, which was subsequently transformed into competent *E. coli* DH5α cells. Plasmid DNA was isolated from selected colonies and sequenced. The plasmid constructs (pRSET-EmGFP and NmIR-GFP-pRSET) were transformed into *E. coli* strains DH5α and BL21(DE3) for measurement of fluorescence as an indication of synA-GFP fusion expression.

### Bacteria cultures

Bacteria were cultured at 37°C with vigorous shaking (250 RPM) in terrific broth. Promoter activity was measured by quantifying the GFP intensity via confocal microscopy and the ImageJ region of interest (ROI) manager tool (15). Five replicates of confocal images per experiment were imported into ImageJ for intensity analysis. Using the ROI manager, a minimum of 2,000 cells were analyzed. In other experiments, fluorescence was measured with a microplate reader. Bacteria cells were inoculated into 96-well plates and grown in LB broth with antibiotics under the same conditions. OD_600_ and fluorescence measurements were taken using the BioTek Synergy H1 microplate reader.

### Transcription factor constructs

To quantify and determine the effects of the TFs on capsule synthesis, the sequences for *crgA*, *lexA*, *misR*, *misS*, *nusG*, and *typA* were retrieved from GenBank based on the sequence of *N. meningitidis* MC58 genome sequence (accession number AE002098.2). The respective TF was amplified via primers designed with a RBS upstream of the ATG initiation codon via the Takara In-Fusion Snap primer design tool. The primers used are listed in Table S2. The amplified products were purified (using the NEB Monarch PCR Cleanup Kit) for directional cloning into the BamH1 site of the pstV28 vector via the Takara cloning kit according to the manufacturer’s recommended procedure. The cloned genes were confirmed via Sanger sequencing.

#### Trans-complementation assay

The genes were transformed into competent NmIR*-GFP-carrying* cells and cells carrying respective deletion mutants of the putative control elements as predicted with software. After confirmation of transformation, the cells were cultured overnight and imaged with confocal microscopy.

#### Confocal microscopy

The transformed bacteria cells were transferred into a µ-Slide 8-Well glass bottom chamber slides and images collected in the LAS X software with a Leica STELLARIS 5 Confocal Microscope. The cells were excited with a 488 nm laser and emission collected at 510 nm.

### Statistical analysis

Imaging data represented three independent biological replicates with five images per sample being used for fluorescence quantitation. The mean fluorescence intensity measurements were determined from the images with the Fiji distribution of ImageJ. Statistical analysis was performed with one-way ANOVA using GraphPad Prism 10 software.

## SUPPLEMENTAL MATERIAL

Tables S1 and S2 spectrum.03301-24-s0001.docx Primers used in the study.

ASM does not own the copyrights to Supplemental Material that may be linked to, or accessed through, an article. The authors have granted ASM a non-exclusive, world-wide license to publish the Supplemental Material files. Please contact the corresponding author directly for reuse.
