# Supplementary material for: Regulation of the promoter for capsular polysaccharide synthesis in Neisseria meningitidis serogroup B by HTH_XRE family transcription factor
Source: Microbiol Spectr. 2025 Apr 24;13(6):e03301-24. doi: 10.1128/spectrum.03301-24 (PMC12131743; doi:10.1128/spectrum.03301-24)
Supplement: Tables S1 and S2 — Primers used in the study. [file spectrum.03301-24-s0001.docx]

**Supplementary Data**

**Table S1: Primers for mutating regulatory elements. The primers used were designed with the NEBaseChanger primer design interface (New England Biolabs).**

| Mutation | Forward primer | Reverse primer |
| --- | --- | --- |
| Up-like element AAAATTTCA | TTTTAAACACAAGCCAAATCC TTA TATAATTATAAATGG | ATATTCGTCACGGATCCGAATTAA TTCCGA |
| Up-like element and -35 region AAAATTTCATTTTAAA | CAC AAG CCA AAT CCT TAT ATA ATT ATA AAT GGC CTA ATT | ATA TTC GTC ACG GAT CCG AAT TAA TTC CGA |
| -10 promoter  TATAAT | TAT AAA TGG CCT AAT TAT AGC ACT TAA TCG AAA | TAA GGA TTT GGC TTG TGT TTA AAA TGA AAT |
| *LexA*  TATATACT | TAT AGA TTC TAA AAT CAT GAA AAG AAT TCT TTG | TAT ATA AGT ATA AGG AAG TTG GAA AGA AGA |
| *rpoD* 16  GAGTATAAT | TAG TAT TCT TCT TTC CAA CTT CCT TAT ACT TAT ATA TAT | TAC GTA CTC ATA AAT TTA TTT CGA TTA AGT GCT ATA ATT |
| *rpoD* 17  TATAGATT | CTAAAATCATGAAAAGAATTCTTTG | AGTATATATATATAAGTATAAGGAAGTTG |
| *rpoD* 18  CTTATACT | TAT ATA TAT ATA CTT ATA GAT TCT AAA ATC ATG AAA AGA ATT CTT | GAA GTT GGA AAG AAG AAT ACT AAT TAT ACT CTA |
| Direct repeat: TATACTTATATATATATACTTA | TAG ATT CTA AAA TCA TGA AAA GAA TTC TTT GCA TTA | AGG AAG TTG GAA AGA AGA ATA CTA ATT ATA CTC |
| 1^St^ Direct repeat:  TATACTTA | AGG AAG TTG GAA AGA AGA ATA CTA ATT ATA CTC | TAT ATA TAT ACT TAT AGA TTC TAA AAT CAT GAA AAG AAT |
| *MisR*  CATTACA | GGTACCAGAGCCGACTTCGGCAAGCTAAAA | CAAAGAATTCTTTTCATGATTTTAGAATCTATAAG |

**Table S2: Primers for amplifying transcription factors. The primers were designed using the In-Fusion Cloning primer design tool (Takara Bio USA, Inc.) based on the genomic sequence of *N. meningitidis* MC58.**

| TF | Forward primer | Reverse primer |
| --- | --- | --- |
| crgA | CGACTCTAGAGGATCGAAGGAGATATACATTTATCCACAG | CGGTACCCGGGGATCATGAAAACCAATTCAGAAGAACTG |
| lexA | CGACTCTAGAGGATCGAAGGAGATATACATTCAATCCAGC | CGGTACCCGGGGATCATGACAATGCACGAGACAACTG |
| misR | CGACTCTAGAGGATCGAAGGAGATATACATTCAGTTTTTG | CGGTACCCGGGGATCATGAGCCGCGTATTACTCG |
| misS | CGACTCTAGAGGATCGAAGGAGATATACATTCAGTTCGC | CGGTACCCGGGGATCATGAAACTGTTCCAACGCATTTTC |
| MisR/misS | CGACTCTAGAGGATCGAAGGAGATATACATTCAGTTCGC | CGGTACCCGGGGATCATGAGCCGCGTATTACTCG |
| nusG | CGACTCTAGAGGATCGAAGGAGATATACATATGTCGAAAA | CGGTACCCGGGGATCTCAGTTAATCTTTTCAACCTGGCTG |
| rpoD | CGACTCTAGAGGATCGAAGGAGATATACATATGTC | CGGTACCCGGGGATCTTACAGCTTGCTGTCTTCGC |
| typA | CGACTCTAGAGGATCGAAGGAGATATACATATGAAACAAA | CGGTACCCGGGGATCTCAATCCAGCTTTTTAAAGTGGCGG |
